# Appropriateness of imaging modality choice by doctors at the Kenyatta National Hospital’s Accident and Emergency Department

**DOI:** 10.4102/sajr.v26i1.2367

**Published:** 2022-06-29

**Authors:** Salman S. Ahmed, Callen K. Onyambu, Eunice Omamo, Alfred Odhiambo

**Affiliations:** 1 Department of Diagnostic Imaging & Radiation Medicine, Faculty of Radiology, University of Nairobi, Nairobi, Kenya; 2 Department of Radiology, Kenyatta National Hospital, Nairobi, Kenya; 3 Department of Radiology, University of Nairobi, Nairobi, Kenya

**Keywords:** imaging appropriateness, imaging guidelines, radiation protection, imaging gently, ionising radiation

## Abstract

**Background:**

Clinical imaging guidelines assist doctors in selecting the most appropriate radiological investigation(s) according to the patient’s clinical presentation and also help to avoid unnecessary radiation exposure.

**Objectives:**

This study aimed to determine the appropriateness of choice of imaging procedures requested by the doctors in the Accident and Emergency Department (A&E) of Kenyatta National Hospital (KNH).

**Method:**

Request forms sent to the KNH Radiology Department from the A&E Department from 01 July 2019 to 31 October 2019 were captured digitally. The request forms were de-identified to ensure confidentiality of patients and requesting doctors. Only the demographic data, clinical summary and radiological examination requested were extracted.

**Results:**

A total of 1053 imaging request forms were captured and analysed using the American College of Radiology (ACR) appropriateness criteria. Adequate clinical summary was provided in 81.3% of the request forms. Appropriate imaging requests were 51.9% whilst inappropriate imaging requests were 34.6%. The clinical scenarios of 13.6% of the imaging requests were not found in the ACR database. Imaging modalities using ionising radiation formed the bulk of the inappropriate investigations at 72.8%. Of these, CT scan had the highest individual inappropriate requests of 49.3%. Only 18.4% of female patients in the reproductive age group had a documented last menstrual period.

**Conclusion:**

Imaging modalities using ionising radiation had the highest percentage of inappropriate radiological requests, especially CT scans requested in the trauma setting. In addition, some clinical scenarios were not captured in the ACR appropriateness criteria, hence the need for local imaging guidelines.

## Introduction

The majority of patients seeking medical care are referred for diagnostic imaging procedures to determine the presence or absence of internal pathologic processes, which are suspected during routine physical examinations.

Modalities such as CT scan, radiography, PET CT, Nuclear Medicine and fluoroscopic procedures use ionising radiation as a method of interrogating the internal organs of the body and use of these modalities has resulted in a 7.1-fold increment in population dose in the United States (US) from 124 000 person-sieverts in 1980 to 880 000 person-sieverts in 2006.^1,2^ A large population based, cohort data linkage study was performed in Australia on 11 million participants to assess the risk of cancer in children and adolescents following exposure to ionising radiation from diagnostic CT scans. Their results indicated a direct correlation between radiation exposure from diagnostic CT scans and cancer incidence. An absolute excess rate for all cancers combined was 9.38 per 100 000 person years at risk and the cancer incidence rate ratio was greater after exposure at early ages.^3^

Each of the given modalities requiring use of ionising radiation has its unique radiation dose.^4,5^ The responsibility lies with the radiologist to reduce exposure as much as possible by ensuring the most appropriate imaging modality is chosen for patient management and by applying the as low as reasonably achievable (ALARA) principle.^6^ The ALARA principle has three main sub-principles, which are justification, optimisation and application of dose limits.^7^

The appropriateness of a radiological test is determined by its ability to provide a solution to a particular clinical scenario, without creating a financial burden to the patient. It should be able to directly impact the clinical management of the patient and consequently be able to improve the net quality of life of the patient.^8,9^

In the West, the need for appropriateness guidelines in radiology came about in the early nineties because of the ever-increasing number of imaging studies requested.^2^ However, no such study has been conducted in East Africa.

The aim of this cross-sectional study was to determine the appropriateness of choice of imaging modality by the doctors at the Kenyatta National Hospital (KNH) Accident and Emergency Department (A&E) and to highlight the level of radiation exposure from inappropriate imaging.

## Methods

A hospital based cross-sectional study was carried out in the KNH Radiology Department from 01 July 2019 to 31 October 2019. All request forms from the A&E were chosen by the principal investigator as they were submitted to the Department of Radiology and digital photographs of the eligible request forms were captured after obtaining consent and de-identifying patient and requesting doctor’s particulars.

The digital photographs with unique identifiers were then transferred to a computer, after which the demographics, clinical summary and radiological investigations requested were analysed and the extracted data were incorporated into a Microsoft Excel data sheet.

The clinical summary of each radiological request form was compared with a similar clinical scenario in the ACR appropriateness criteria database (https://acsearch.acr.org/list), and the corresponding radiological exam requested was compared with that suggested in the appropriateness criteria for that clinical scenario. This was evaluated solely by the principal investigator.

### Ethical considerations

The study was approved by the Kenyatta National Hospital/University of Nairobi Ethics and Scientific Review Committee (Reference number: KNH-ERC/A/184 and approval number P257/04/2019). All the participants signed an informed consent form before participating in the study.

## Results

In this study a total of 1053 imaging request forms were analysed using ACR appropriateness criteria of which 856 forms had adequate clinical information (included primary complaint(s), duration of complaint(s) and exact anatomical location of the complaint(s) based on clinical exam) whilst 197 forms had inadequate clinical information (one worded, no duration of complaint(s) or anatomical location provided). Of these, slightly over half, 546 (51.9%, 95% confidence interval [CI]: 48.8% – 54.9%) were appropriate whilst 364 (34.5%, 95% CI: 31.7% – 37.5%) were inappropriate. A small percentage of the requests (143/1053, 13.6%) were not captured in the ACR appropriateness criteria.

Amongst the appropriate imaging requests, ultrasound was the highest (63.6%), whilst amongst the inappropriate imaging requests, CT scan had the highest percentage (49.1%) as tabulated in Table 1.

**TABLE 1:** Appropriateness of imaging modality choice by the doctors at Kenyatta National Hospital Accident and Emergency department based on the American College of Radiology appropriateness criteria.

Modality	Appropriate according to ACR appropriateness criteria		Inappropriate according to ACR appropriateness criteria		Not captured In the ACR appropriateness criteria		Total
	*n*	%	*n*	%	*n*	%	
Ultrasound	262	63.6	98	23.8	52	12.6	412
X-ray	198	47.8	154	37.2	62	15.0	414
CT scan	85	37.8	111	49.3	29	12.9	225
MRI	1	50.0	1	50.0	0	0.0	2

ACR, American College of Radiology.

A comparison was made between the inappropriate request forms requiring use of ionising radiation (41.4%, 95% CI: 37.6% – 45.2%) and those not requiring ionising radiation (23.9%, 95% CI: 19.8% – 28.0%), which showed a statistically significant difference (*p* < 0.0001). This was calculated based on the data presented in Table 2.

**TABLE 2:** A comparison of the appropriateness of imaging choice of ionising and non-ionising radiation.

Type of imaging modality	Appropriate	Inappropriate
Ionising radiation (CT and X-rays)	283	265
Non-ionising radiation (ultrasound and MRI)	263	99

**Total**	**546**	**364**

## Discussion

This study sought to establish the appropriateness of imaging and modality choice by the doctors at the KNH A&E department. This is the first study evaluating imaging appropriateness guidelines in the country.

The majority of request forms (81.3%) provided adequate clinical information. However, this is slightly lower than a similar study conducted in Nigeria, which revealed that 86.9% of the analysed request forms had adequate clinical information.^7^

Plain radiography (X-rays) was the most frequent (39.3%) examination requested by the doctors at the KNH A&E department, followed closely by ultrasound (39.1%). The CT scan imaging requests amounted to 21.4% of the total whilst MRI requests were low at 0.2% because of unavailability of the MRI scanner during the study period. The two imaging modalities utilising ionising radiation formed the bulk of the total imaging requests at 60.7% of the total.

Overall appropriateness of imaging and modality choice by the KNH A&E doctors was 51.9%. This was lower than a score of 71.0% by residents working in emergency medicine in a similar study performed in the US.^10^ However, there are no local benchmarks to compare with. As basic radiology training is provided to all undergraduate students, national benchmarking can easily be performed. This shows a need for improvement in selecting the most appropriate imaging modality by the KNH A&E doctors.

The total number of inappropriate imaging request forms was 364. Of these, a staggering 73% (265/364) of the imaging requests, involved X-rays and CT scan imaging, with resultant exposure to unnecessary ionising radiation. According to imaging modalities, inappropriate requests for CT scan were the highest at 49.3%, whilst inappropriate requests for X-rays were 37.2%. As previously discussed, any exposure to ionising radiation carries a risk of developing cancer.^11^ Deterministic effects are dose dependent and can be controlled, however, stochastic effects are dose independent and have no threshold dose. Patients need to be protected from unnecessary radiation exposure to prevent stochastic effects of radiation exposure.

Computed tomography had the highest (49.3%) individual proportion of inappropriate choice of imaging modality. A substantial majority were requested in the trauma setting. Many of the request forms did not have an appropriate clinical indication for the scan, for example, head CT scan requests in the setting of mild head trauma. Routine head CT scan delivers a radiation dose of 2.1 mSv and is associated with a lifetime risk of cancer of 0.23 per 1000 patients.^12^ The CT radiation doses of the other body parts are much higher and so is the lifetime risk of developing cancer.^12^

One of the postulated reasons for the increased indiscriminate use of imaging modalities using ionising radiation by the requesting doctors is lack of knowledge of the radiation doses delivered by the various imaging modalities and the detrimental effects of radiation exposure.^13^ Its knowledge is vital for selecting the correct imaging modality study and communicating the same to patients and relatives so that they can make an informed decision. Unnecessary radiation exposure not only harms patients but also leads to increased population exposure. There needs to be a local regulatory board that continuously measures the environmental dose to the population, so that appropriate measures can be put in place.^14^

In this era of increased legal proceedings against medical practitioners, the practice of defensive medicine is on the rise. This has led to increased costs of healthcare as doctors are playing safe and not making judgement on clinical basis alone, but increasingly requesting laboratory and radiological investigations to secure a diagnosis.^15^ This could be an important contributory factor to the overutilisation of CT scan and X-rays.

Lack of national and in-hospital imaging guidelines could also be an important additional factor in choosing inappropriate imaging. A pinned up clinical algorithm in the A&E Department, which outlines and guides the doctor on when to, for example, request for a head CT scan in trauma would go a long way in reducing inappropriate requests.^3^ Having a radiologist approving and protocoling requests of high radiation dose imaging modalities such as CT would also help in reducing their numbers.^3^

There were some clinical scenarios that were not listed in the ACR criteria and consequently the most appropriate imaging and modality choice for those clinical scenarios could not be determined. This contributed to 13.6% of the total imaging requests. The most frequent clinical scenario involved penetrating injuries to various body regions, apart from penetrating injury to the back with suspected injury to the ureters. This highlights the need to have local guidelines to address this deficiency and one based on local prevalence of diseases.

For women in the reproductive age group, 15–49 years,^16^ the doctors had poor compliance with writing the date of the last menstrual period (LMP) on the radiology request form. Only 18.4% of imaging request forms of female patients within the reproductive age group had a documented LMP. Prior knowledge of the LMP is of importance when ordering radiological examinations involving ionising radiation. The deterministic dose for inducing malformation is 100 mGy, which is above the value used in diagnostic imaging. However, there is no safe dose to prevent stochastic effects in the embryo that would lead to cancer occurrence later in life. The commonest cancer secondary to in utero radiation exposure is leukaemia.^17,18^

The negative impact of inappropriate ultrasound studies comes about in the form of increased financial burden and increased turnaround times for patients. Imaging appropriateness guidelines not only help in preventing unnecessary radiation exposure but also reduce financial strain and waiting times for patients.

The radiologist is an essential member of the clinical team who assists with a diagnosis. To accomplish this, the radiologist needs the imaging request form to be duly completed in its entirety. This includes patient’s demographic data, dating the request form, LMP for female patients in the reproductive age, adequate clinical summary, provisional diagnosis, underlying comorbidities and whether previous examination(s) have been performed. Apart from LMP, as discussed here, the doctors at the A&E department are for the larger part, attempting to complete the form in its entirety. A similar study performed in Nigeria showed that less than 20.0% of the forms had adequately detailed clinical information, compared with 81.3% at the KNH A&E Department.^7,19^

## Limitations

Unavailability of MRI scan within KNH during the study period resulted in inadequate information on its appropriateness as a choice of imaging modality. The MRI requests were taken to private institutions, which offered MRI scans.

## Conclusion

The highest number of inappropriate radiological requests involved imaging modalities using ionising radiation. Of these, CT scan, especially those requested in the trauma setting, had the highest proportion of inappropriate choice. The doctors were most proficient in providing adequate clinical information as seen in the majority of the request forms but fell markedly short in documenting the LMP for female patients within the reproductive age group. The inability to determine the appropriateness of some of the imaging requests has highlighted the need for establishing local guidelines based on local disease prevalence.

## Recommendations

- Well displayed imaging algorithms within the A&E would enable the doctors to make split-second decisions on the need for imaging and in choosing the most appropriate imaging modality, and if uncertain, discussion with the attending radiologist may help to select the most appropriate imaging modality.
- For women within the reproductive age group who do not have a documented LMP date on the request form, imaging utilising radiation should be deferred, unless benefits outweigh risks.
- Continuous education on the harmful effects of radiation exposure and radiation dose delivery per modality chosen should be provided to all clinicians requesting the various imaging modalities. This would enable them in making informed decisions after carefully weighing the merits and demerits of individual imaging modalities.
- There is need for national imaging guidelines, which are tailored to the prevalence of local diseases.
